# Hyperparameter Optimization of a Convolutional Neural Network Model for Pipe Burst Location in Water Distribution Networks

**DOI:** 10.3390/jimaging9030068

**Published:** 2023-03-14

**Authors:** André Antunes, Bruno Ferreira, Nuno Marques, Nelson Carriço

**Affiliations:** 1 Sustain.RD, Escola Superior de Tecnologia de Setúbal, Instituto Politécnico de Setúbal, 2914-508 Setúbal, Portugal; 2 NOVA LINCS, Department of Computer Science, Faculdade de Ciências e Tecnologia, Universidade NOVA de Lisboa, 2829-516 Caparica, Portugal; 3 INCITE, Escola Superior de Tecnologia do Barreiro, Instituto Politécnico de Setúbal, 2839-001 Lavradio, Portugal

**Keywords:** convolutional neural networks, deep learning, hydraulic model, hyper parameterization, pipe burst location

## Abstract

The current paper presents a hyper parameterization optimization process for a convolutional neural network (CNN) applied to pipe burst locations in water distribution networks (WDN). The hyper parameterization process of the CNN includes the early stopping termination criteria, dataset size, dataset normalization, training set batch size, optimizer learning rate regularization, and model structure. The study was applied using a case study of a real WDN. Obtained results indicate that the ideal model parameters consist of a CNN with a convolutional 1D layer (using 32 filters, a kernel size of 3 and strides equal to 1) for a maximum of 5000 epochs using a total of 250 datasets (using data normalization between 0 and 1 and tolerance equal to max noise) and a batch size of 500 samples per epoch step, optimized with Adam using learning rate regularization. This model was evaluated for distinct measurement noise levels and pipe burst locations. Results indicate that the parameterized model can provide a pipe burst search area with more or less dispersion depending on both the proximity of pressure sensors to the burst or the noise measurement level.

## 1. Introduction

Leakage in water distribution networks (WDN) necessarily implies the inefficient use of water resources, of energy for water pumping and treatment, and of chemicals in water treatment plants, whilst also posing a health risk to the population due to risks of bacteria and pollutant contamination [1]. The first step towards efficient leakage management is the accurate assessment of the water volume that is lost. To this end, WDNs are usually divided into smaller district metered areas (DMA), in which the flowrates are continuously measured in the area’s inlets and outlets and the consumed water volume is periodically measured in consumers. Leakage volumes in DMA are usually assessed through the minimum night flow (MNF) regime [2,3]. During the MNF, consumption from users is usually minimum and the dominant consumption is due to leakage. Such MNF analysis can reduce the search area from the whole WDN to a particular DMA. Nonetheless, additional burst and leak location techniques are necessary to find the approximate location inside the DMA and the exact location at the street level. This is relevant for burst events which are not visible at the street level and that can go unreported for months [4].

Distinct software-based techniques have been developed in recent decades for leak detection and location in WDNs. These techniques can be roughly divided into model-based and data-driven methods. Data-driven methods [5,6,7] use monitoring data that, combined with tools such as data mining or artificial intelligence algorithms, allow the identification of possible leak location zones. Although these methods do not require deep knowledge regarding the WDN’s hydraulic characteristics (e.g., pipe characteristics or individual demands), extensive historical data records of monitoring data and precise information on past pipe burst events are necessary. In many water utilities, service work orders are collected by operational technicians who register imprecise and incomplete data, which directly compromises the implementation and efficiency of such techniques [8]. Thus, the application of such methods is limited to burst detection, which often fails to successfully locate bursts in the WDN [5].

Model-based methods use hydraulic simulation models and detect and locate leaks based on the comparison of numerical results with measurements from pressure sensors and flowrate meters. Examples of these methods are inverse analysis [9,10,11], network sensitivities’ computation and analysis [12,13], error-domain model falsification [14,15], and that based on a classification problem [16,17,18].

The development of machine learning techniques in recent years has enhanced the use of classification algorithms for locating pipe bursts. In essence, leakage scenarios are obtained by hydraulic simulation and the corresponding pressure measurements are obtained. In a second stage, such scenarios (and corresponding measurements) are used to train a classifier. After a burst event has occurred, the observed pressure values can be processed, and the trained classifier can be used to infer the burst location. In this way, Soldevila et al. [16] firstly generated data for all possible leak locations, to which synthetic Gaussian noise was added. Then, nodes were grouped by the effect that a burst event (in each location) has on the pressure sensors. Finally, the K-nearest neighbor classifier is trained and used for classification. In a distinct approach, Zhang et al. [17] firstly used K-means clustering and nodal pressure sensitivities to divide the network into K different zones. Leakage scenarios for each zone were obtained by using hydraulic simulation and by adding a random leakage demand to junctions selected by Monte Carlo simulations. The training samples are finally used to train a multiclass support vector machine classifier. More recently, Hu et al. [18] used density-based spatial clustering of applications with noise (DBSCAN) to divide the network into a number of zones; such zones are used as learning labels for the classification problem, which is solved using three distinct classifiers, namely, support vector machines, a naïve Bayes classifier, and K-nearest neighbor. Without considering node grouping, Zhou et al. [19] used a fully linear DenseNet to extract features in pressure patterns, whilst Javadiha et al. [20] used convolutional neural networks to learn from pressure residuals. More recently, Romero et al. [21] transformed pressure vectors into images using the Gramian angular field image encoding procedure, thus transforming the problem into an image classification problem. An hyperparameter study was carried out by Kim et al. [22] for fine-tuning the artificial neural network (ANN) for pipe burst detection, but this neither used a full real WDN network nor aimed at locating the burst in the specific WDN pipe. Although promising results were obtained using these classification methods, the parameters’ selection is often a black box, and not much information is provided on how the parameters could be optimally tuned for other case studies with distinct characteristics. Neural networks, in particular, have multiple configurable hyperparameters, and their parameterization is of upmost importance. There is, however, a lack of guidelines for defining the optimal hyperparameters from which water utilities can directly benefit. Such guidelines would allow the use and adequate parameterization of such techniques for detecting and location of pipe bursts in their WDNs, directly contributing to the much-needed implementation of such systems in real water distribution systems.

The current paper has two main objectives: the first is to explore different neural network parameterizations specifically tailored for the pipe burst location problem. The problem is applied to a real case study located in the Lisbon metropolitan area of Portugal. An extensive analysis of classification results for distinct combinations of hyperparameters is performed; the variable hyperparameters include the termination criteria, the training dataset size, the need (or not) for normalization, the training batch size, the regularization in the optimizer, and the neural network structure. Secondly, the paper assesses the effect of measurement noise and uncertainty in pipe burst locations by using a classification-based method with an optimal set of parameters. To the authors’ knowledge, this is an innovative approach, aiming to help water utilities in developing and tuning their own models.

The main novel contributions of this research work are (i) the summary of lessons learned in the tuning process of neural networks for the pipe burst location problem; (ii) the extensive testing of method’s performance, considering distinct measurement noise levels; and (iii) the demonstration of the analysis using a real case study with more than one thousand nodes.

## 2. Methods

### 2.1. Pipe Burst Events in Water Distribution Networks

Pressure and flow rate data play an important role in the daily operation of water distribution networks. Such data carry information about the actual condition and performance of these systems, and can be used in different frameworks of hydraulic engineering and water resources analysis, namely for pipe burst location.

A pipe burst causes an increase in the flowrate along the main flow paths from the sources to the burst location, thus resulting in higher water consumption than expected. A generalized pressure drop is thus experienced in the water distribution network, which is captured by the installed pressure sensors. This creates a more distinct signature in pressure and flowrate sensors than a burst-free situation, and this can be used to infer the location of the pipe burst event using distinct techniques.

### 2.2. Locating Pipe Bursts by Classification Using Neural Networks

**Figure 1:**
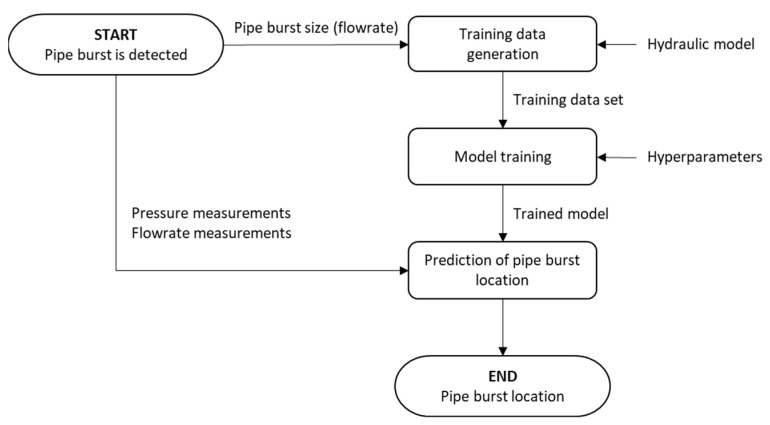
Flowchart of proposed methodology for pipe burst location using neural networks.

The current subsection presents the method for locating pipe burst events by classification using neural networks. It is assumed that the pipe burst event was already detected, and its size (i.e., burst flowrate) is known; this may be achieved, for instance, by minimum night flow analysis or by using data-driven techniques. A flowchart of the proposed methodology is present in Figure 1 and is composed of three main steps: (1) training data generation; (2) model training; and (3) prediction of the pipe burst’s location. The following sections further detail each step of the proposed methodology.

#### 2.2.1. Training Data Generation

It is difficult to obtain real data related to pipe burst events in water distribution networks that is adequate for model training. On one hand, pipe burst events are not that common, so they may not be representative of the diversity of possible pipe burst locations and sizes. On the other hand, and when such data exist, they might not reflect the network’s characteristics (e.g., network layout, demand patterns, valve settings).

Recent innovations in hydraulic modeling, namely the integration of both real-time measurements of water consumption in end users and valves and pump’s settings, are allowing water utilities to simulate the hydraulic behavior of the water distribution network under normal operation, with a relatively small uncertainty between computed and measured values [23,24]. During a pipe burst event, such models can be used to simulate distinct burst conditions in the network, thus generating training data.

In this study, data generation is performed by simulating pipe burst scenarios (e.g., 25% of the total inlet flowrate) in distinct network locations, namely at all network nodes or in a reduced-space set of nodes. Each pipe burst is computed as follows:

(1) $$Q=CP^{\alpha },$$

where *Q* is the leak flowrate, *C* is the emitter coefficient representing the burst orifice effective area, *P* is the pressure-head, and *α* is the pressure exponent which depends on the pipe material and geometry of the orifice, herein considered equal to 0.5 [25].

Each pipe burst scenario leads to a set of pressure and flowrate simulated values (at sensor locations) and is related to a specific label (containing the identification of the burst location). So, *k* datasets of pressure and flowrate (and a similar number of classification labels) are obtained. Note that at this point, each label contains a single set of measurements (or a single observation), which may affect the performance of the classifier. The following procedure is used to increase the number of observations for each label and to account for the uncertainty in model parameters and metering errors: (1) the maximum perceptual error between the simulated (using the hydraulic model) and real measured values should be estimated for a burst-free situation (e.g., 3%); (2) *r* copies of each label’s dataset are generated by introducing random uniform noise in each measurement of ± the maximum perceptual error. Note that real-world WDN measurements always present uncertainty, with noise, missing values, or outliers. The noise introduced in the datasets also tries to model this kind of data in a more realistic way.

The generated training datasets are then used to train the neural network model, as explained in the next chapter.

#### 2.2.2. Model Training

**Figure 2:**
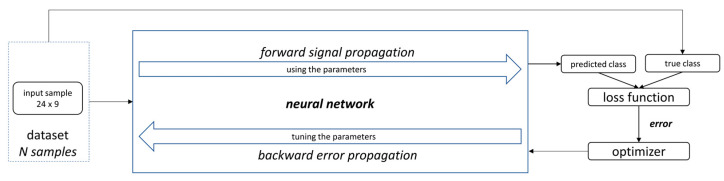
Neural network training.

Training a neural network consists of determining the weights of each neuron so that when presented with a specific input, it produces the expected output. The training process happens in two stages: forward signal propagation and reverse error propagation. A backpropagation algorithm (BP) calculates the gradient of a loss function with respect to the weights of the network. BP uses gradient descent to search the space of possible hypotheses in an iterative way, reducing the error when fitting the network to the training examples. Gradient descent converges to a local minimum error in training regarding the network weights. The training error must be a differentiable function of the hypothesis parameters. BP can create meaningful representations of the input data. The internal layers of a multilayer neural network learn intermediate features’ representation, which is useful for learning the target function and is not explicit in the network inputs [26]. The training process is visualized in Figure 2.

In this study, the training of the model is supervised, meaning that all training set samples are labelled. Each training sample is composed of a matrix of sensor measurements and is associated with a classification label of a specific node in the hydraulic simulation model (which represents a real location). In sum, the model receives the training data as input and uses it for model training as previously described, using forward signal and backward error propagation in order to produce a trained data model. The trained model can then be used to predict a classification when fed with a new data sample. The model output is a vector with the burst location probability for each relevant network node.

Common issues with training are underfitting and overfitting of models. Underfitting occurs when the model still has the opportunity to learn, and has poor performance even on the training data. Overfitting occurs when the model cannot generalize new data but still has good performance on the training data. Neural network models trained with small datasets tend to overfit. Strategies to prevent overfitting include:

(1) Data augmentation by training with more data. Using more data for training usually produces more accurate models, but the more data that are used adds training time overhead that must be considered in the scope of the desired task. If the increased training duration does not provide relevant loss and accuracy gains in the desired training time frame, it should be avoided. So, it is relevant to determine when to continue and when to stop adding more training data.
(2) Reducing the complexity of the network model by limiting the number of parameters and features.
(3) Weight regularization can be viewed as a forced simplification of the model. Several parameters can be used when applying regularization, including early stopping, dropout, and L1 and L2 coefficients’ regularization.
(4) Using dropout layers helps to prevent model overfitting. The dropout procedure relies on randomly omitting hidden units with a certain probability [27].
(5) Batch normalization allows training to be faster and more stable [28].

An optimized combination of some of these strategies is usually effective in guaranteeing an adequately trained model that avoids both under and overfitting.

#### 2.2.3. Prediction of Pipe Burst Location

**Figure 3:**
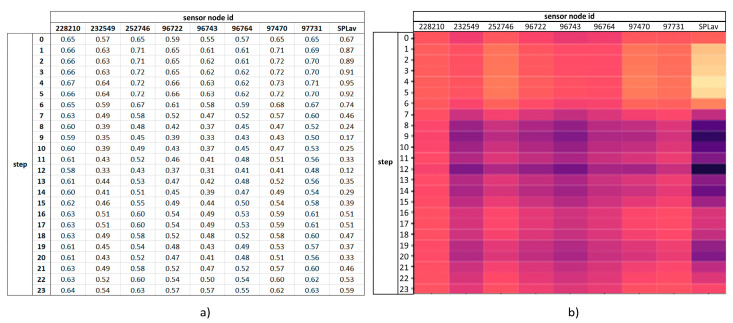
Example of a data sample, showing the normalized hourly measurements during a 24 h period (one day) for each WDN sensor: (**a**) numeric values in tabular form; and (**b**) visual representation using a color scale.

The trained model can be used to predict a pipe burst location, given a new sample dataset. In a real-world scenario, a set of measurements from the sensors placed in the network would be provided after pipe burst detection. This set consists of both pressure and flow rate measurements during a given period of time (e.g., past 24-hourly measurements), and can be normalized before using as an input to the model. A set of measurements is depicted in Figure 3, both as numeric values in tabular form and as a visual representation using a color scale. In real-life applications, key assumptions related to hydraulic modelling must be guaranteed, namely that the hydraulic WDN model would have to be calibrated and updated to reflect the actual network configuration and hydraulic behavior.

### 2.3. Investigation on the Hyper Parameterization

**Figure 4:**
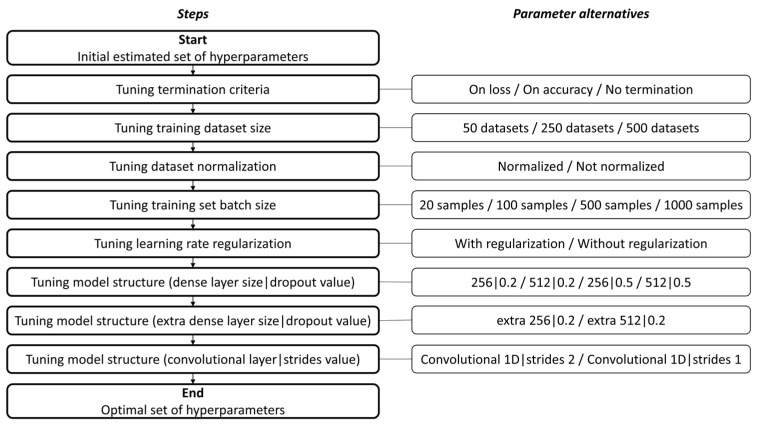
Sequence of steps to determine the optimal set of hyperparameters, including the tuning of training termination criteria, dataset size, dataset normalization, training set batch size, learning rate regularization and model structure (dense layer size and dropout layer value, adding an extra dense layer, and adding a convolutional layer with a specific strides value).

The classification problem requires the configuration of several hyperparameters to achieve optimal results. A sequence of tuning steps is proposed to assess the optimal set of hyperparameters for the model, as displayed in Figure 4.

The first parameter to be tuned is the early stopping termination criteria. Early stopping allows the termination of training based on some predefined criteria (e.g., loss or accuracy). The parameter can be configured based on a minimum delta or an epoch patience size (occurring after that predefined number of epochs without progress on the monitored metric). The direction of the monitored metric (increasing or decreasing) can also be used.

The second tuned parameter is the dataset size, which determines the number of data samples of each location available for training the model.

The third tuned parameter is data normalization. The normalization process consists of converting the actual measurement to a value between 0 and 1. Normalization is carried out by taking in account measurements relating to each sensor and normalizing using the minimum and maximum limits from all measurements in all datasets relating to that sensor.

The fourth parameter is the training set batch size, which is the number of data samples processed in each epoch step.

The fifth tuned parameter is the use (or not) of optimizer learning rate regularization.

The sixth and last tuned parameter is the model structure. Several configurations are tested, with a distinct number of dense layers and variable number of units. Convolutional layers and pooling are also tested.

The tuning process is performed by considering an initial set of parameters to be able to define a baseline. Furthermore, some criteria must be established:

- The number and location of the pressure and flow sensors that will be used to monitor a possible pipe burst in the network.
- The magnitude of the pipe burst that defines the size of the burst that is simulated at each node.
- Monitoring metrics, which are used to assess the evolution of the training process by evaluating the monitored values at each training epoch step.
- The measurement noise level, which is used to model the uncertainty present in real-world hydraulic systems.
- The use (or not) of network clustering or other techniques to reduce the search space (or, in other words, the number of possible outputs of the classification problem).
- The number of time steps in the hydraulic simulation.
- The training dataset split for training and validation, which is a threshold that defines the number of data samples that are used for training and for validation of the network.
- The training model optimizer, which is the algorithm that updates the weights and learning rate of the model to reduce loss and improve accuracy. In this study, the Adam optimizer algorithm is used.

The following tuning process is carried out for each parameter (i.e., in each step of Figure 4):

(1) Multiple alternatives are considered for the parameter being tuned (e.g., a termination criteria based on loss or accuracy). The remaining parameters should be considered as in the initial set or, if available, the tuned values.
(2) The model, using each alternative parameter, is trained and tested.
(3) The best parameter alternative is chosen, and the next parameter can be tuned.

Each test is used to tune the model, using the best value for each hyperparameter.

## 3. Application: Results and Discussion

A computational application was developed for this research, using Python 3.8.2 as the programming language. The TensorFlow 2.9.1 (TF) package was used to develop the neural network, and the TensorBoard 2.9.1 plugin was used for assessment and visualization of training results [29]. Hydraulic simulations are carried out in EPANET [30], which is a water distribution system modeling software package developed by the United States Environmental Protection Agency, using the Water Network Tool for Resilience 0.4.2 (WNTR) Python package [31].

### 3.1. Case Study Description

**Figure 5:**
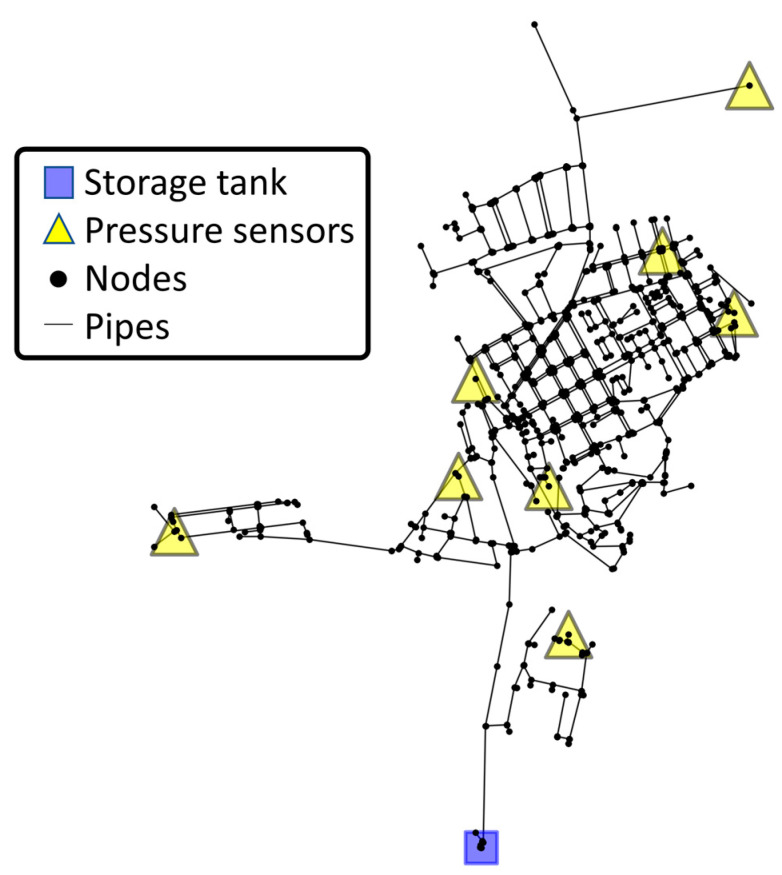
Case study of WDN displaying monitoring sensors and inlets.

The case study uses an existing WDN in the Lisbon metropolitan area, serving a population of 14,000 inhabitants and supplying approximately 700,000 m^3^/year of drinking water. The network supplies a 4 km^2^ area containing residential, commercial, and services buildings such as supermarkets, schools, a central hospital, a large sports complex, shops, and industrial plants, it being very heterogeneous in terms of construction typology and functionality. The WDN has about 2200 service connections and an approximate length of 36 km. The pipe material is 63% asbestos cement, 34% polyvinyl chloride (PVC), and 3% high-density polyethylene (HDPE), with diameters in the range of 32 to 400 mm. The EPANET hydraulic model for this network includes 967 pipes and 1262 nodes, with one storage tank, simulated as a constant level reservoir, and an associated pumping station operating at a constant pressure throughout the day. The hydraulic model includes 24 h consumption patterns, allowing extended period simulations. The WDN can be visualized in Figure 5, showing the storage tank which contains the flow rate meter (using a blue square marker) and the location of the eight pressure sensors (using a yellow triangle marker). The process used for determining the optimal number and location of pressure sensors for WDN monitoring is presented in [4,32].

### 3.2. Hyperparametrization

**Figure 6:**
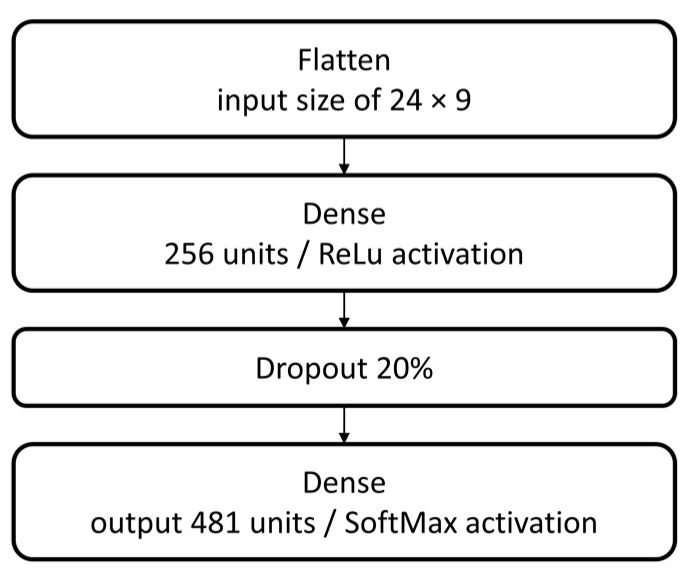
Neural network model.

A simple neural network structure is implemented as a baseline for starting the tests procedure. This structure consists of a group of sequential layers: flatten input with a shape of 24 rows by 9 columns, dense with 256 units and rectified linear unit (ReLu) activation, a 20% dropout, and dense output with 481 units and SoftMax activation, as displayed in Figure 6.

An initial set of estimated parameters is chosen as the baseline for training the neural network (See Table 1). The burst size considered for training data generation and prediction is 20% of the total inlet flow rate. The hydraulic simulation consists of 24 hourly time steps. No network clustering is used, but an initial search space reduction for removing valve nodes is carried out; thus, 481 relevant WDN nodes are considered.

Training data are generated by simulating a burst at every each WDN node during 24 hourly timesteps. The measurement noise used when generating the training dataset is considered equal to 0.0001% (practically zero or no noise). The effect of measurement noise level on the model’s performance is later assessed in Section 3.3. A dataset split of 70% is used for training, and the remaining 30% samples are used for validation.

The model optimizer is Adam, with learning rate regularization. Adam uses a computational efficient method with low memory requirements, and is adequate for big data problems with lots of parameters [33].

The metrics to use when assessing loss and accuracy are directly dependent on the neural network structure. In this study, since SoftMax is used as activation of the output layer, sparse categorical cross entropy and sparse categorical accuracy metrics are used for monitoring loss and accuracy, respectively (the sparse categorical variants of the metrics are used due to integer conversion of categories before training the model).

The categorical cross entropy can be calculated as follows:

(2) $$CE=-\sum_{i=1}^{n}y_{i}\cdot \log{\hat{y}}_{i}\;,$$

where n is the number of classes, $y_{i}$ is the truth value of the real class, and ${\hat{y}}_{i}$ is the probability of the inferred class.

The categorical accuracy can be calculated as follows:

(3) $$CA=\frac{TP+TN}{N},$$

where N is the number of observations, TP is the number of true positives, and TN is the number of true negatives.

#### 3.2.1. Effect of Termination Criteria

The early stopping (ES) functionality in training can be useful for preventing excessive training and possible overfitting of models, but if misconfigured can stop training at a local minimum and prevent the model from further learning. A test was performed to determine if ES should be considered to stop training based on some predefined criteria (e.g., loss or accuracy). ES was configured to stop after 20 epochs without metric progression. Using ES with a patience setting of 20 epochs and monitoring for validation loss, the model stopped training at epoch 335, achieving a final validation loss and accuracy of 1.796 and 41.5%, respectively (Table 2, C1). Using the same setting but monitoring for validation accuracy, the model stopped the training at epoch 98, achieving a final value for the validation loss and accuracy of 2.071 and 35.5%, respectively (Table 2, C2). Training for the full 5000 epochs without ES achieved a final validation loss and accuracy of 1.591 and 46.63%, respectively (Table 2, C3).

**Figure 7:**
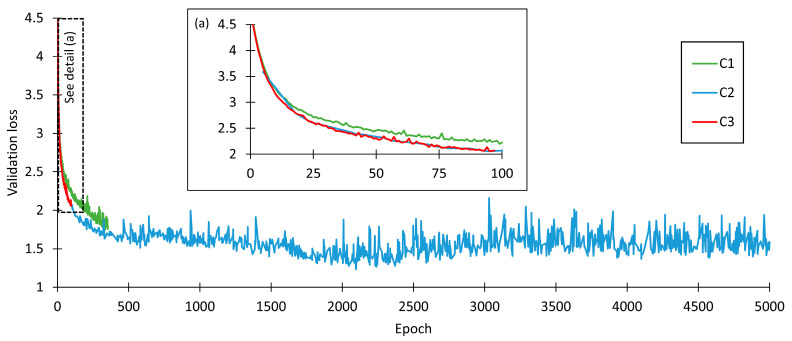
Effect of termination criteria. (**a**) Zoomed detail for the dash-delimited area, highlighting the early stopping of C2 parameter combination.

The full training (5000 epochs) allowed the detection of the inflection of the validation loss and accuracy curves, showing that the best values were achieved just before epoch 2500, as can be seen in Figure 7. ES criteria with a patience setting of 20 epochs were too low to achieve the optimal values. Taking in account the minor difference between the optimal value and the final value at 5000 epochs, and that, in this specific case, the fine tuning of the other parameters demonstrates that the early stopping parameter stops being useful, a decision was made to stick to training until 5000 epochs were reached. This decision will also allow full assessment of the validation loss curve progression.

#### 3.2.2. Effect of Training Dataset Size

The number of datasets available for neural network training is a relevant factor. Three distinct sizes for dataset generation were devised: 50, 250, and 500. Each dataset consists of the sensor measurements after placing a simulated burst in each of the relevant network nodes throughout the 24 hourly timesteps. So, for the current case study (a network with 481 relevant nodes after SSR and 9 sensors), 50 datasets consist of 24,050 samples, 250 datasets consist of 120,250 samples, and 500 datasets consist of 240,500 samples.

Training the model with 50 datasets achieved a validation loss and accuracy of 1.992 and 36.0%, respectively, in 1 h and 34 min (Table 3, C4); using 250 datasets for training achieved a validation loss and accuracy of 1.667 and 41.1%, respectively, in 8 h (C5), and training with 500 datasets achieved a validation loss and accuracy of 1.591 and 46.6%, respectively, in 17 h (C6). The best result was achieved with the training using 500 datasets, but the extra amount of time needed is relevant, considering that it might limit the method’s usability in near real-time applications and there was only a little gain achieved in validation accuracy. Thus, 250 datasets are used for the remaining tests.

#### 3.2.3. Effect of Dataset Normalization

Two distinct sets of training data consisting of 250 datasets were generated. The first set was normalized between 0 and 1. The second set was not normalized. Training with 250 normalized datasets achieved a validation loss and accuracy of 1.667 and 41.1%, respectively (Table 4, C7), whilst training with non-normalized datasets resulted in a validation loss and accuracy of 6.176 and 1.5%, respectively (Table 4, C8). These results demonstrate the relevance of and need for normalization.

#### 3.2.4. Effect of Training Batch Size

The batch size determines the number of samples used to calculate one step in the training process. Several steps are performed during one epoch, and the number of steps is calculated as the amount of training samples divided by the batch size. Using a batch size of 20 achieved a validation loss and accuracy of 5.16 and 1.4%, respectively; this was interrupted at epoch 1000 after lasting for around 8 h, thus making the training too long (Table 5, C9). Training with a batch size of 100 achieved a validation loss and accuracy of 1.584 and 46.1%, respectively; this lasted around 8 h for 5000 epochs (Table 5, C10). A batch size of 500 samples achieved a validation loss and accuracy of 1.15 and 61.1%, respectively, with a duration of around 2 h for 5000 epochs (Table 5, C11). A batch size of 1000 achieved a validation loss and accuracy of 1.463 and 55.2%, respectively, for 1000 epochs, thus performing worst and being interrupted after 19 min (Table 5, C12). In sum, a greater batch size can reduce the training duration, but the performance depends on the available system memory. Thus, the best result was found using a training batch size of 500 samples.

#### 3.2.5. Effect of Optimizer Learning Rate Regularization

Configuring the optimizer with learning rate regularization (using inverse time decay with a decay rate of epoch steps multiplied by 1000) achieved a validation loss and accuracy of 1.021 and 75.4%, respectively (Table 6, C13). The same training without learning rate regularization achieved a validation loss and accuracy of 1.15 and 61.1%, respectively (Table 6, C14). Therefore, the best result was achieved using optimizer learning rate regularization.

#### 3.2.6. Effect of Neural Network Structure

The neural network topology and structure are very relevant parameters for obtaining good results. The optimal structure is usually obtained after experimentation, and there is no known formula as it depends mostly on the specific problem. This study uses dense layers and convolutional layers as building blocks for a neural network. A simple network structure was devised as a baseline for all previous tests, using a dense layer with 256 units, followed by a 20% dropout layer, both of which achieve a validation loss and accuracy of 1.021 and 75.4%, respectively (Table 7, C15). Distinct structure variations were tested to find the best solution. Using a dense layer with 512 units followed by a 20% dropout layer achieved a validation loss and accuracy of 0.872 and 79.9%, respectively (Table 7, C16). A structure with a dense layer with 256 units and a 50% dropout layer achieved a validation loss and accuracy of 1.567 and 66.8%, respectively (Table 7, C17), whilst a dense layer with 512 units and a 50% dropout layer achieved a validation loss and accuracy of 1.194 and 77.2%, respectively (Table 7, C18). The best structure was found with C16, since this combination presented both the best validation accuracy and loss.

Sometimes multiple layers can be combined to achieve better results. Thus, we trialed adding an extra dense and dropout layer to the previous test’s best configuration (C16). A dense layer with 512 units and 20% dropout layer followed by another dense layer with 256 units and 20% dropout layer achieved a validation loss and accuracy of 3.771 and 35.6%, respectively (Table 8, C19). A dense layer with 512 units and 20% dropout layer followed by another dense layer with 512 units and 20% dropout layer achieved a validation loss and accuracy of 5.871 and 19.6%, respectively, being interrupted after 1000 epochs (Table 8, C20). Based on these results, it was concluded that adding these extra layers did not provide better results, and therefore the configuration from C16 was kept as the basis of the subsequent tests.

A neural network structure configuration using a convolutional layer was tested. This configuration consists of the best previous dense configuration (C16), this time preceded by a convolutional 1D layer followed by a global max pooling layer, which is then connected to dense layer with the number of units equal to the relevant 481 network nodes and a SoftMax activation, building a final model with five hidden layers. So, using a convolutional layer with 32 filters, a kernel size of 3 and strides equal to 2 achieved a validation loss and accuracy of 0.401 and 88.1%, respectively (Table 9, C21). A configuration using the same parameters but with strides equal to 1 achieved a validation loss and accuracy of 0.3274 and 90.3%, respectively (Table 9, C22). Based on these results, it is concluded that the best configuration is C22, with strides equal to 1.

In sum, the first tuning consisted of establishing the layer size and dropout value. The second tuning consisted of adding extra dense layers to the base configuration. Adding these extra layers did not provide better results, and therefore the configuration from C16 was kept as the basis of the next tests. As a third tuning of this hyperparameter, an explicit convolutional layer was added before the classification stage (CNN with the better dense classifier model). This convolutional layer extracts relevant features from the input data. This configuration provided better results than using a simple classifier. The fine tuning of the strides parameter in the convolution can provide an attenuation in the data. Using a higher value for strides seemed to discard relevant information, providing worst results.

**Figure 8:**
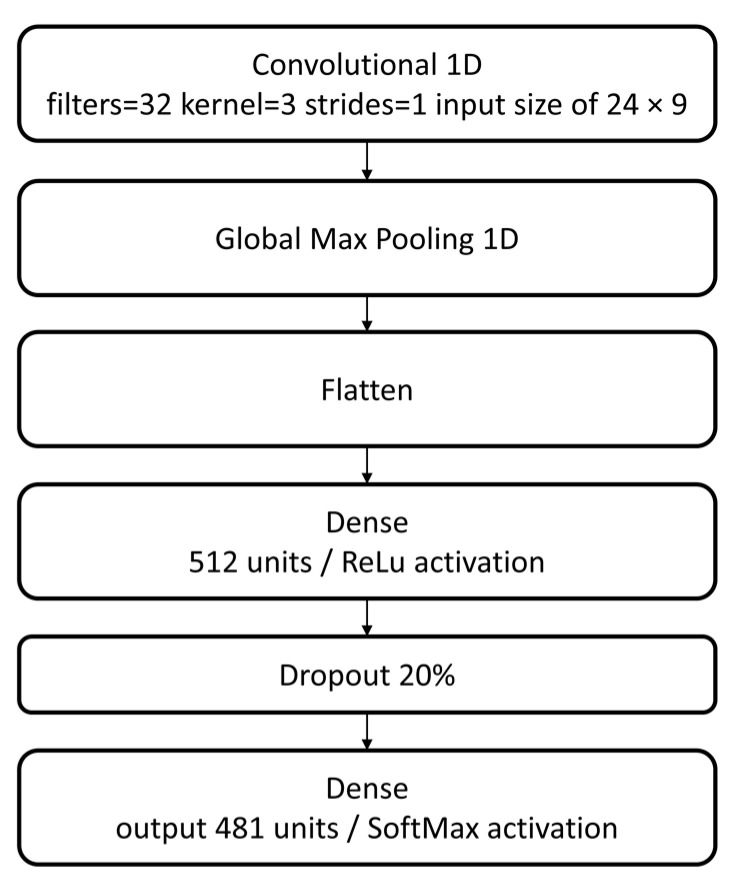
CNN model configuration.

The C22 configuration, which provided optimal results and was thus selected as the final model configuration, can be visualized in Figure 8. It consists of training a model configuration based on a CNN with a convolutional 1D layer (using 32 filters, a kernel size of 3 and strides equal to 1) for a maximum of 5000 epochs, using a total of 250 datasets (using a [0, 1] data normalization with tolerance equal to max noise) and a batch size of 500 samples per epoch step, and optimized with Adam using learning rate regularization.

**Figure 9:**
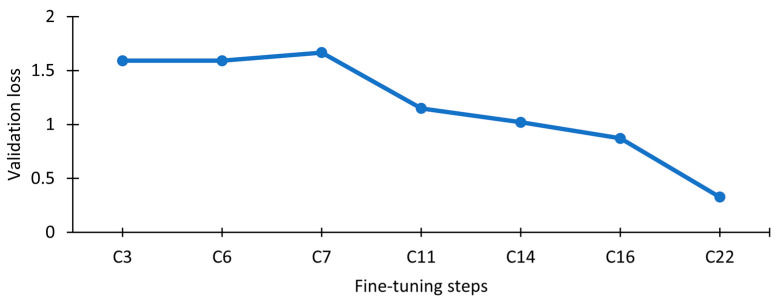
Evolution of the validation error during the hyperparameter fine-tuning process.

The evolution of the validation loss during the overall hyperparameter fine-tuning process can be visualized in Figure 9.

#### 3.2.7. Inference Test

After the model is trained, it can be used for inference of pipe burst locations. To assess the model’s inference consistency, a test was carried out by simulating a pipe burst in four network nodes located in distinct places throughout the WDN (i.e., by generating pressure and flow rate measurements associated with a pipe burst in four distinct locations). The result of each inference is the probability of the burst being located in each node. Based on these results, two metrics are used: the predicted distance and the weighted distance. The predicted distance consists of the Euclidean distance between the real pipe burst location and the node with the highest probability (herein referred as the inferred node). The weighted distance is the weighted average between each node’s probability and the Euclidian distance to the real pipe burst location. The mean values after ten location predictions for a pipe burst in the four nodes are displayed in Table 10.

### 3.3. Study on the Effect of Measurement Noise in Pipe Burst Location

To study the effect of measurement noise on pipe burst location, in order to take into account the uncertainty of real measurements, two additional training sets of 250 datasets were created, the first one using a noise value of 1% and the second using a noise value of 3%. Training the model with the 0% noise dataset provided a validation accuracy and loss of 90.3% and 0.3274, respectively (Table 11, C23). Training the model using the 1% noise dataset achieved a validation loss and accuracy of 2.092 and 33.7%, respectively (Table 11, C24). The use of a measurement noise level of 3% in the training dataset achieved a validation loss and accuracy of 3.015 and 19.2%, respectively (Table 11, C25).

As expected, the increase in measurement noise level leads to a decrease in the validation accuracy and increase in validation loss. Nonetheless, note that the WDN topology is important when assessing the method results. Although this is a classification problem in that it can be either correct or wrong, in practice, selecting an incorrect location a few meters away from the real target (in a network with dozens of km) cannot be deemed wrong. The accuracy of 19.22% (with 3% noise) can seem rather small from a classification point of view, but as is further demonstrated in this chapter, it can be concluded that the most probable nodes are near the true location. This is the reason that the maps provided with each inference are so important. In practice, water utilities aim to estimate the approximate location at a street level using these computational methods. Based on this approximate location, more precise methods can be used, such as acoustic equipment or valve-step testing.

**Figure 10:**
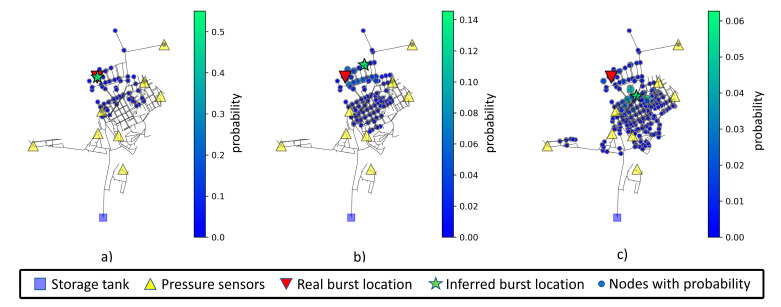
Network prediction areas for node 20, considering (**a**) 0% noise level, (**b**) 1% noise level, and (**c**) 3% noise level. Nodes with higher probability are displayed in a greener color and with lower probability in a bluer color, following the scale presented to the right of each chart.

To assess model inference consistency using noisy datasets, measurements were made for inferences using 0%, 1%, and 3% noise. Each inference is made with a newly generated sample not available in training or validation datasets. The mean values after ten location predictions of a pipe burst located at node 20 are displayed in Table 12. The increase in measurement noise level leads to an increase in both mean weighted and predicted distances. Note that node 20 is in the upper left section of the network and is far from any sensor. The inferred node and associated area of interest for node 20 can be viewed in Figure 10. The visualization depicts the possible burst areas (nodes where there is a probability greater than zero). The most relevant nodes in these areas are displayed with a larger size and a greener color, following the scale presented to the right of each chart.

The increase in measurement noise level leads to an increase in nodes with assigned probability (a greater blue area) and nodes with higher probability (a higher number of green nodes).

**Figure 11:**
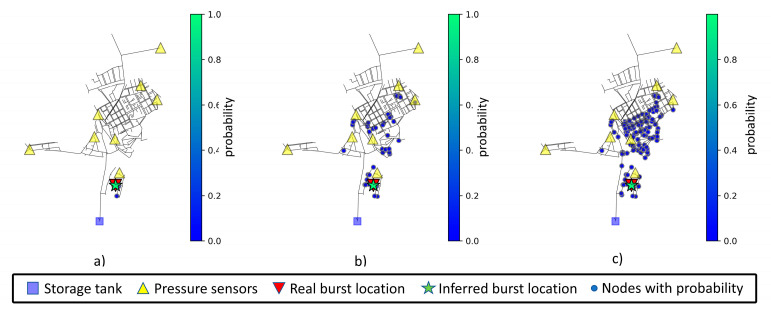
Network prediction areas for node 40, considering (**a**) 0% noise level, (**b**) 1% noise level, and (**c**) 3% noise level. Nodes with higher probability are displayed in a greener color and with lower probability in a bluer color, following the scale presented to the right of each chart.

The mean values after ten location predictions for a pipe burst located at node 40 are displayed in Table 13. Interestingly, the increase in measurement noise level does not increase the weighted or predicted distances. Node 40 is in the bottom right section of the network and in a very specific and isolated area with a nearby sensor. The inferred node and associated area of interest for node 40 can be viewed in Figure 11.

**Figure 12:**
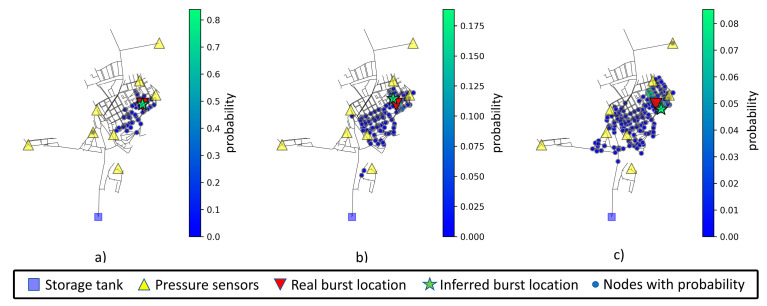
Network prediction areas for node 235, considering (**a**) 0% noise level, (**b**) 1% noise level, and (**c**) 3% noise level. Nodes with higher probability are displayed in a greener color and with lower probability in a bluer color, following the scale presented to the right of each chart.

The mean values after ten location predictions for a pipe burst located at node 235 are displayed in Table 14. The increase in measurement noise level leads to an increase in both mean weighted and predicted distances. Note that node 235 is in the middle right section of the network, with no nearby sensor. The inferred node and associated area of interest for node 235 can be viewed in Figure 12.

**Figure 13:**
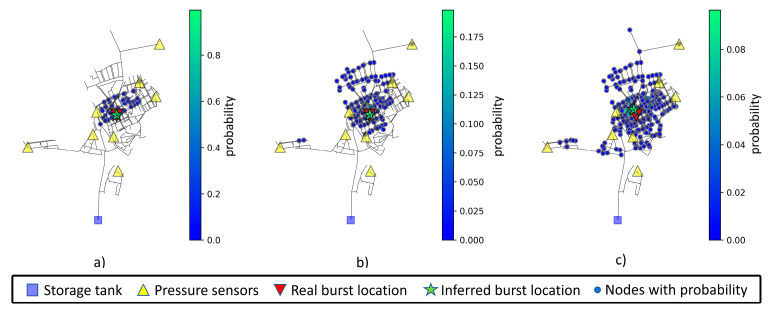
Network prediction areas for node 285, considering (**a**) 0% noise level, (**b**) 1% noise level, and (**c**) 3% noise level. Nodes with higher probability are displayed in a greener color and with lower probability in a bluer color, following the scale presented to the right of each chart.

Finally, the mean values after ten location predictions for a pipe burst located at node 285 are displayed in Table 15. Once again, the increase in measurement noise level leads to an increase in both mean weighted and predicted distances. Node 285 is in the middle of the network, with no nearby pressure or flow rate sensors. The inferred node and associated area of interest for node 285 can be viewed in Figure 13.

In sum, the model starts to have difficulty in accurately predicting the burst location as higher measurement noise levels are considered. Whilst without uncertainty, the model is almost always accurate, with predictions in a distance range between 0 and 5 m, the added noise level makes the model more prone to a certain degree of inaccuracy. Nevertheless, it is still capable of making a valuable prediction, and provides an area of interest which can be further reduced using hardware-based techniques.

With 1% noise added, the area of interest (composed by nodes that have a probability greater than zero) starts to become more dispersed and, depending on the proximity of the burst node to a sensor, the model can still be accurate. Even if the model is inaccurate, the set of relevant nodes (with highest probability) highlight the possible area in which the burst occurs. The distance range for prediction of the analyzed nodes is between 0 and 200 m.

With 3% noise added, the area of interest tends to grow and spread, with the probabilities being much lower in magnitude, as can be observed in Figure 10, Figure 11, Figure 12 and Figure 13. The weighted distance and prediction distance grow, with the distance range for prediction of the analyzed nodes falling between 7 and 202 m.

Compared to existing classification methods, which often cluster the WDN into zones prior to the classification problem, the proposed method considers each possible burst location individually and, as such, the region of interest is composed of nodes with a given assigned probability. This ensures that the inference result is not affected by the clustering procedure.

Furthermore, many of the existing model-based methods for pipe burst location fail to consider the uncertainty in model parameters and the errors in measurement readings. As such, when the uncertainty is significant, those methods can pinpoint to completely wrong locations. Ferreira et al. [34] compared three distinct model-based techniques for this same case study and concluded that given high uncertainty levels (e.g., noise level of 3%), the unique inferred location is often quite distant from the true location (e.g., between 200 and 300 m). This unique location is not an ideal result since, if wrongly inferred, the water utility may be looking for a pipe burst where there is not one. On the other hand, the proposed method provides a region of interest which varies in size according to the expected uncertainty level. For a water utility, this region of interest is a much more reliable result, since this region can be further reduced in the field, for instance, by operating valves (e.g., step testing), and by assessing if the inlet flow rate increases.

## 4. Conclusions

This study presented a hyper parameterization optimization process for a convolutional neural network (CNN) specifically applied to pipe burst location in water distribution networks (WDN). By assessing flow rate and pressure measurements, the trained model can infer the location of a pipe burst in a WDN by specifying the exact node (using the node with highest probability) or an associated area of interest (considering the individual probability of each node). The effect of uncertainty in measurements was also studied in order to assess the model’s reliability in a real-world scenario. A set of guidelines for model hyper parameterization was also devised, allowing water utilities to tune the hyperparameters of their own models. The suggested optimal neural network model configuration proved to be reliable and accurate, providing promising prediction results for situations of no noise or a noise level of 1%. For high measurement noise levels (3%), the burst search area tends to grow and spread, with the probabilities being much lower in magnitude.

In future works, this method can be adapted to include input information at the week level, for instance, by using more steps (e.g., 24 h × 7 days), or the input information of special days such as holidays. On both situations, a new hyperparameter optimization process would be required to adapt the model to each specific case. Additionally, it can be assessed if the optimal parameters vary or remain the same depending on the case study and on the input information (weekly, daily, or single day).

## Figures and Tables

**Table 1:** Initial set of estimated parameters.

Parameter	Initial Estimation
Network sensors	8 Pressure and 1 Flow
Burst size	20% of inlet flow rate
Timesteps	24 hourly time steps
Clustering	No
Search space reduction (SSR)	Yes, reducing from 1262 to 481 nodes
Loss Metric	Sparse Categorical Cross Entropy
Accuracy Metric	Sparse Categorical Accuracy
Noise size	0.0001%
Train percentage	70%
Optimizer	Adam

**Table 2:** Termination criteria results.

	C1	C2	C3
Termination criteria	Early stopping validation loss (patience 20)	Early stopping validation accuracy (patience 20)	5000 epochs
Validation accuracy	0.4152	0.3551	0.4663
Validation loss	1.796	2.071	1.591
Training accuracy	0.3052	0.2766	0.3355
Training loss	2.242	2.423	2.045
Finish epoch	355	98	5000

**Table 3:** Number of datasets results.

	C4	C5	C6
Number of datasets	50	250	500
Validation accuracy	0.3609	0.4115	0.4663
Validation loss	1.992	1.667	1.591
Training accuracy	0.3026	0.3171	0.3355
Training loss	2.208	2.14	2.045
Finish epoch	5000	5000	5000

**Table 4:** Dataset normalization results.

	C7	C8
Normalization	Normalized between 0 and 1	Without normalization
Validation accuracy	0.4115	0.0155
Validation loss	1.667	6.176
Training accuracy	0.3171	0.0217
Training loss	2.14	6.181
Finish epoch	5000	5000

**Table 5:** Batch size results.

	C9	C10	C11	C12
Batch size	20	100	500	1000
Validation accuracy	0.0141	0.4612	0.6113	0.5522
Validation loss	5.16	1.584	1.15	1.463
Training accuracy	0.0139	0.314	0.5075	0.4719
Training loss	5.507	2.108	1.357	1.634
Finish epoch	1000	5000	5000	1000

**Table 6:** Learning rate regularization results.

	C13	C14
Learning rate regularization	With regularization	Without regularization
Validation accuracy	0.7543	0.6113
Validation loss	1.021	1.15
Training accuracy	0.5894	0.5075
Training loss	1.3	1.357
Finish epoch	5000	5000

**Table 7:** Layer size and dropout percentage results.

	C15	C16	C17	C18
ANN structure	size256 + dropout0.2	size512 + dropout0.2	size256 + dropout0.5	size512 + dropout0.5
Validation accuracy	0.7543	0.7995	0.6689	0.7726
Validation loss	1.021	0.872	1.567	1.194
Training accuracy	0.5894	0.6558	0.3231	0.4379
Training loss	1.3	1.063	2.251	1.773
Finish epoch	5000	5000	5000	5000

**Table 8:** Extra dense layer results.

	C19	C20
ANN structure	C16 + size256 + dropout0.2	C16 + size512 + dropout0.2
Validation accuracy	0.3565	0.1965
Validation loss	3.771	5.871
Training accuracy	0.6578	0.5599
Training loss	0.9424	1.22
Finish epoch	5000	1000

**Table 9:** Convolutional layer strides parameter tuning results.

	C21	C22
ANN structure	conv1D strides 2	conv1D strides 1
Validation accuracy	0.8816	0.9035
Validation loss	0.4017	0.3274
Training accuracy	0.8506	0.8514
Training loss	0.3401	0.4049
Finish epoch	5000	5000

**Table 10:** Inference mean values for nodes 20, 40, 235, and 285.

Node	20	40	235	285
Mean weighted distance (m)	2.701	0.0	3.897	0.314
Mean predicted distance (m)	1.78	0.0	0.0	0.0

**Table 11:** Burst location results considering distinct measurement noise levels.

	C23	C24	C25
Noise	0%	1%	3%
Validation accuracy	0.9035	0.3374	0.1922
Validation loss	0.3274	2.092	3.015
Training accuracy	0.8514	0.4124	0.29
Training loss	0.4049	1.887	2.585
Finish epoch	5000	5000	5000

**Table 12:** Inference mean values for node 20, with noise.

	Noise		
	0%	1%	3%
Mean weighted distance (m)	2.70	152.26	225.57
Mean predicted distance (m)	1.78	200.08	201.66

**Table 13:** Inference mean values for node 40, with noise.

	Noise		
	0%	1%	3%
Mean weighted distance (m)	0.0	0.0	13.64
Mean predicted distance (m)	0.0	0.0	7.04

**Table 14:** Inference mean values for node 235, with noise.

	Noise		
	0%	1%	3%
Mean weighted distance (m)	3.90	71.73	138.91
Mean predicted distance (m)	0.0	64.75	104.75

**Table 15:** Inference mean values for node 285, with noise.

	Noise		
	0%	1%	3%
Mean weighted distance (m)	0.31	105.83	221.70
Mean predicted distance (m)	0.0	91.97	164.27

## Data Availability

The data presented in this study are available upon request from the corresponding author. The data are not publicly available due to being obtained from third parties.
